# Supplementary material for: Topological dislocation modes in three-dimensional acoustic topological insulators
Source: Nat Commun. 2022 Jan 26;13:508. doi: 10.1038/s41467-022-28182-2 (PMC8791950; doi:10.1038/s41467-022-28182-2)
Supplement: Supplementary file 1 — Supplementary Information [file 41467_2022_28182_MOESM1_ESM.pdf]

Supplementary Information for

"Topological dislocation modes in three-dimensional acoustic topological insulators"

Liping Ye,[1] Chunyin Qiu,[1*] Meng Xiao,[1*] Tianzi Li,[1] Juan Du,[1] Manzhu Ke,[1*] and Zhengyou Liu[1,2*]

[1]Key Laboratory of Artificial Micro- and Nano-structures of Ministry of Education and School of Physics and Technology, Wuhan University, Wuhan 430072, China

[2]Institute for Advanced Studies, Wuhan University, Wuhan 430072, China

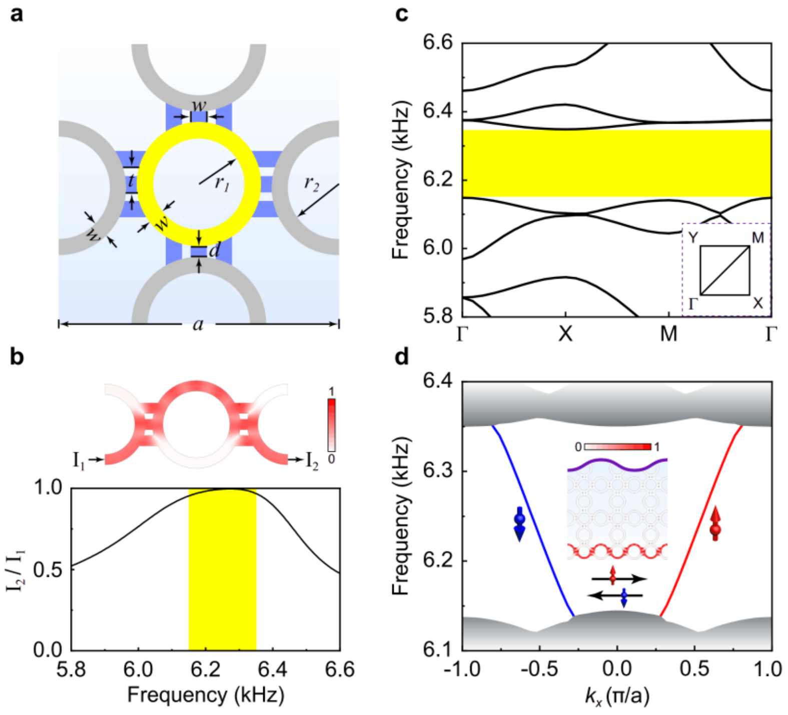

Supplementary figure 1 Construction of the 2D acoustic analog of QSH insulator. a, Geometry of the unit cell. For a square array of the unit cells, the site ring-waveguides (yellow) are coupled through equidistant straight tubes (blue) and coupler ring-waveguides (gray). The structure parameters are: $a = 208$ mm, $r_1 = 34$ mm, $r_2 = 38$ mm, $w = 12$ mm, $d = 8$ mm, and $t = 18.5$ mm. The straight tubes and coupled ring-waveguides (collectively dubbed as in-plane couplers) are optimized to attain strong coupling but negligible pseudospin flip. All the waveguides have a constant thickness $h = 10$ mm for the experiments below. **b**, Top panel: Numerical setup used for analyzing the coupling strength of the in-plane coupler, where the lateral half rings correspond to site ring-waveguides and the middle ring represents a coupler ring-waveguide. In the case of strong coupling, the sound signal correlated with pseudospin-up will excite a pressure amplitude concentrating at the upper half coupler ring-waveguide, as exemplified at by the simulation at 6.27 kHz (color). Bottom panel: Power transmission spectrum that characterizes the coupling strength induced by the in-plane coupler. Specifically, the power transmission $\mathrm{I}_2/\mathrm{I}_1 > 0.95$ spans over the interested nontrivial band gap in **c**, indicating that almost all of the incident waves reach the desired outlet without flipping the pseudospin. **c**, Bulk band structure of the acoustic analog of QSH insulator. The nontrivial band gap (ranging from 6.15 kHz to 6.35 kHz) is highlighted by yellow. **d**, Band structure simulated for a ribbon periodic in the *x* direction and finite in the *y* direction. For clarity, here we provide only the spin-momentum locked edge modes localized at the lower boundary (color lines), where the gray shadows are projected bulk bands. Inset: Eigenfield distribution exemplified at 6.27 kHz, which is the same for both pseudospins since they are related by time-reversal symmetry.

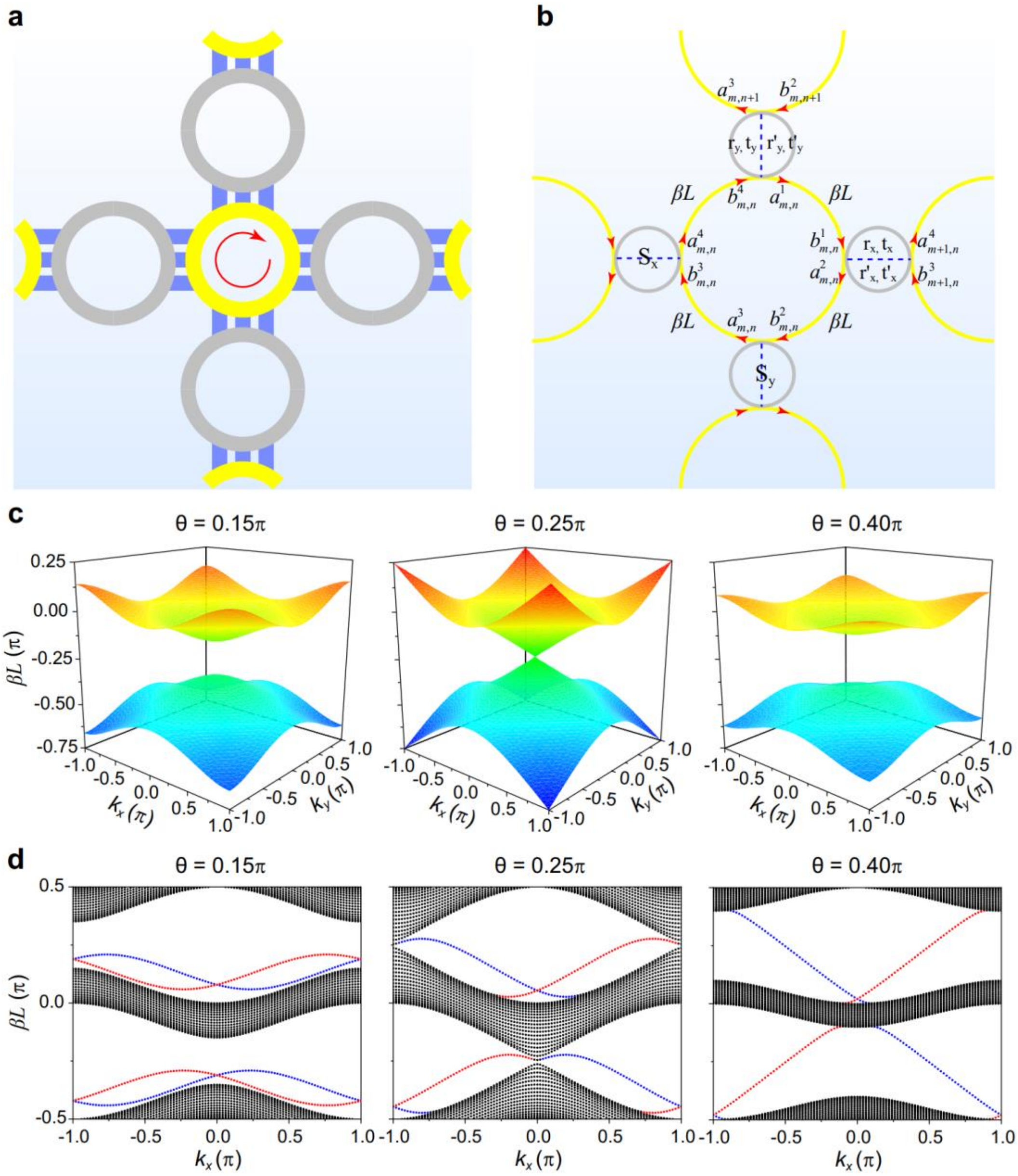

Supplementary figure 2 Transfer matrix method for the coupled ring-waveguides. a, **b**, The coupled ring-waveguides (**a**) and the corresponding equivalent model (**b**). As proved in Refs. [1-4], this 2D structure becomes a topological insulator when the coupling between adjacent waveguides is sufficiently strong. This topological nontrivial phase cannot be captured with a simple tight-binding analysis (typical for weak couplings while here we deal with strong couplings between waveguides) and thus, the transfer matrix method is adopted to analyze this 2D system[1-4]. Here we focus on the pseudospin-down (denoted by the red arrow in **a**) and assume no pseudospin flipping. The response of pseudospin-up can be derived similarly. Let (m, n) denotes the lattice site and $\left|\varphi_{m,n}\right\rangle = \left[a^1_{m,n}, a^2_{m,n}, a^3_{m,n}, a^4_{m,n}, b^1_{m,n}, b^2_{m,n}, b^3_{m,n}, b^4_{m,n}\right]$ represents the corresponding amplitudes of acoustic waves in the four quarters of the site ring-waveguides. The amplitudes $\left|a_{m,n}\right\rangle = \left|a^1_{m,n}, a^2_{m,n}, a^3_{m,n}, a^4_{m,n}\right\rangle$ and $\left|b_{m,n}\right\rangle = \left|b^1_{m,n}, b^2_{m,n}, b^3_{m,n}, b^4_{m,n}\right\rangle$ are related by $\left|a_{m,n}\right\rangle = e^{-i\beta L}\left|b_{m,n}\right\rangle$. Here βL

describes the phase delay across each quarter of the ring waveguide and is frequency dependent in a practical system, L is the length of each quarter of the site ring-waveguide and β is the propagation constant which is also dumbed as Floquet eigenvalues or "quasi-energies" in the band structure calculation[1-7]. Here, we will refer to βL as the "quasi-energy" for simplicity. The wave amplitudes at sites (*m*, *n*), (*m+1*, *n*) in the *x* direction, and (*m*, *n+1*) in the *y* direction are related by $\left(a^4_{m+1,n}, a^2_{m,n}\right) = S_x\left(b^1_{m,n}, b^3_{m+1,n}\right)$ and $\left(a^3_{m,n+1}, a^1_{m,n}\right) = S_y\left(b^4_{m,n}, b^2_{m,n+1}\right)$, where $S_x = \left(r_x, t'_x; t_x, r'_x\right)$ and $S_y = \left(r_y, t'_y; t_y, r'_y\right)$ are both assumed to be frequency independent for simplicity, and r_x, r'_x, t_x, t'_x represent the coupling between waveguides in the *x* direction, while r_y, r'_y, t_y, t'_y represent the coupling between waveguides in the *y* direction. In this system, the couplings conserve energy and are assumed to be the same $S_x = S_y = \left(\sin\theta, i\cos\theta; i\cos\theta, \sin\theta\right)$, where θ characterizes the strength of coupling between adjacent site ring-waveguides. For a periodic system, the band structure is governed by $\mathrm{e}^{-4i\beta L} + 2iY\mathrm{e}^{-2i\beta L} - 1 = 0$, where $Y = -\frac{1}{2}\sin 2\theta\left(\cos k_x + \cos k_y\right)$, and k_x (k_y) is the Bloch wave vector in the *x* (*y*) direction. Thus, the two bulk bands are $\beta_+ L = m_+\pi + \sin^{-1} Y/2$ and $\beta_- L = m_-\pi + (\pi - \sin^{-1} Y)/2$, where m_+ (m_-) is an integer. **c** plots the band structures for θ = 0.15π, 0.25π, and 0.4π, where we can see the band gap closes at 0.25π. As proved in Refs. [1-4], $\theta = 0.25\pi$ is the topological phase transition point, and across which the system turns from topologically trivial phase ($\theta <$ 0.25π) into the topological insulator phase ($\theta > 0.25\pi$). This topological phase transition can also be convincingly observed in the projected band diagram in **d**, which considers a strip of coupled ring-waveguides with infinite sites in the *y* direction and periodic in the *x* direction. Here the black dots represent bulk states, and the red and blue dots represent edge states on the upper and lower edges of the strip geometry, respectively. For $\theta = 0.15\pi < 0.25\pi$, the system is a conventional insulator, and each edge hosts two edge states with positive as well as negative group velocities at different K_x. For $\theta = 0.40\pi > 0.25\pi$, the system is a topological insulator, and the gapless edge states span the nontrivial band gaps. The above analysis verifies that the coupled waveguide system is topologically nontrivial within the strong coupling regime when $\theta > 0.25\pi$. In a real system, θ is frequency dependent and can be retrieved as $\theta = \arcsin(\sqrt{I_2/I_1})$ where the power transmission I_2/I_1 obtained by the manner shown in the supplementary Fig. 1b, and the nontrivial band gap region is obtained by the manner shown in supplementary Fig. 1.

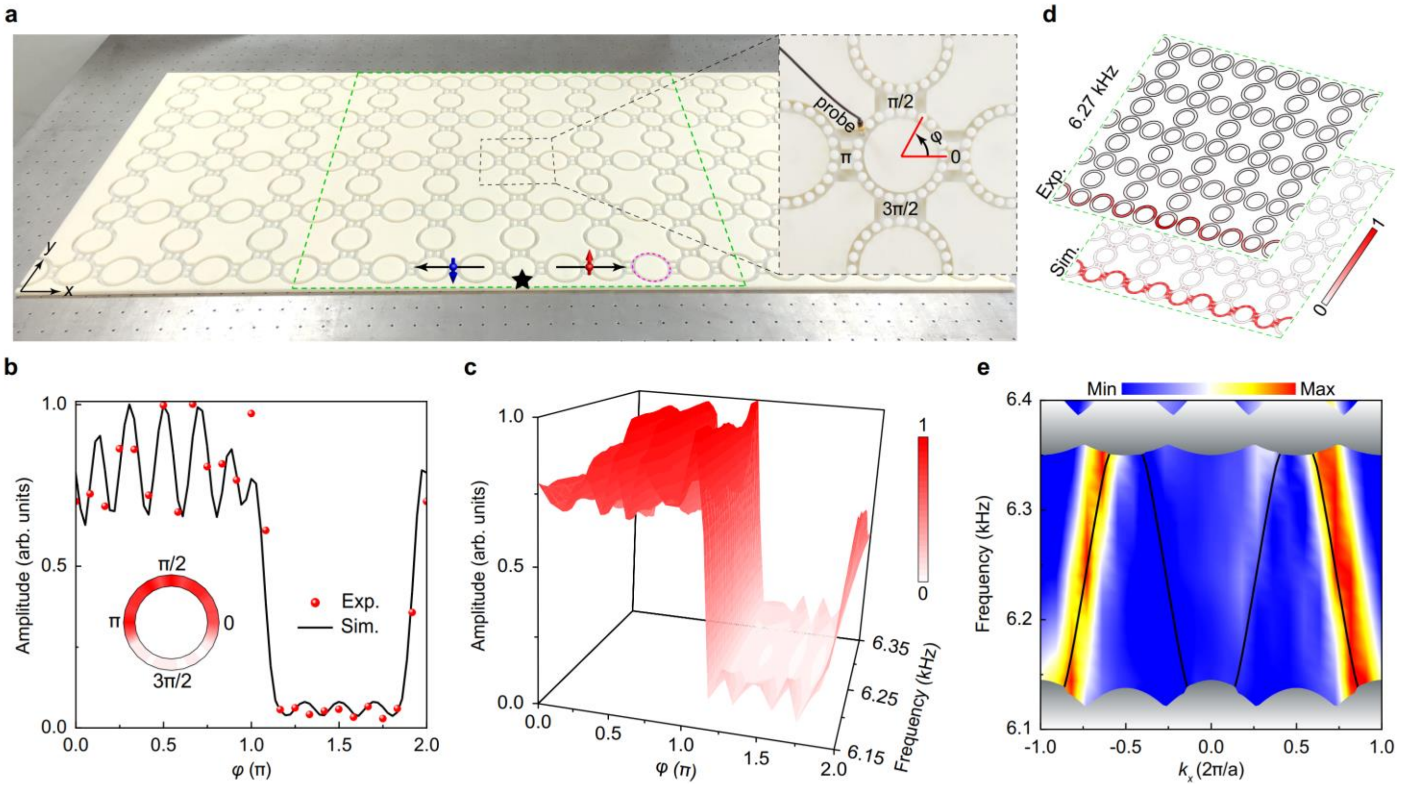

Supplementary figure 3 Experimental characterizations for our 2D acoustic analog of QSH insulator. **a**, Experimental setup. The black star labels the position of the point-like sound source. During the measurements, the sample is covered with a transparent acrylic plate, which is perforated with small holes for inserting the sound source and probe. Inset: Zoom-in photograph that demonstrates the sound detection, where φ labels the azimuthal angle in the ring-waveguide. **b**, φ-dependent pressure amplitude distribution measured in a coupler ring-waveguide (highlighted in **a** with the magenta dashed circle) at 6.27 kHz. The experimental data (red spheres) capture well the simulation (black line). Inset: A direct visualization of the experimental data inside the ring. Consistent with the top panel in supplementary Fig. 1b, the sound energy for the rightward-moving (pseudospin-up) mode concentrates in the upper half coupler ring-waveguide, which is a direct evidence for the strong coupling induced by the in-plane coupler. **c**, Similar to **b**, but plotted from 6.15 kHz to 6.35 kHz, which confirms the strong coupling over the nontrivial bulk gap. **d**, Experimental field pattern (top layer) scanned at 6.27 kHz inside the green dashed box sketched in **a**, comparing with the associated simulation result (bottom layer). The experimental result agrees well with the simulation, except for the attenuation induced by the unavoidable loss. **e**, Measured dispersion of the topological edge modes (bright color), which reproduces excellently the simulation result (black lines). Again, the gray shadows represent the numerical bulk-band projections. Note that the Fourier component of the measured edge modes in the second Brillouin zone is much stronger than that exhibited in the first Brillouin zone.

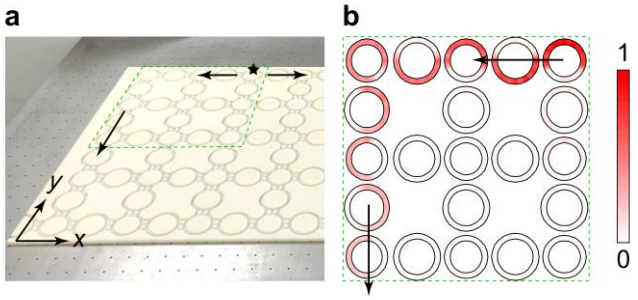

Supplementary figure 4 Testing topological robustness of the helical edge modes in the 2D acoustic analog of QSH insulator. a, Photograph of the experimental setup. The black star marks the position of the sound source and the black arrows indicate the propagating directions of the excited topological edge modes. **b**, Pressure amplitude distribution scanned in the region highlighted by the green dashed box in **a**. Again, the field profile is exemplified at 6.27 kHz. It shows that the topological edge modes bypass the $90°$ bending corner smoothly without visible scattering into the bulk, as an evidence of the topological robustness against the boundary deformation.

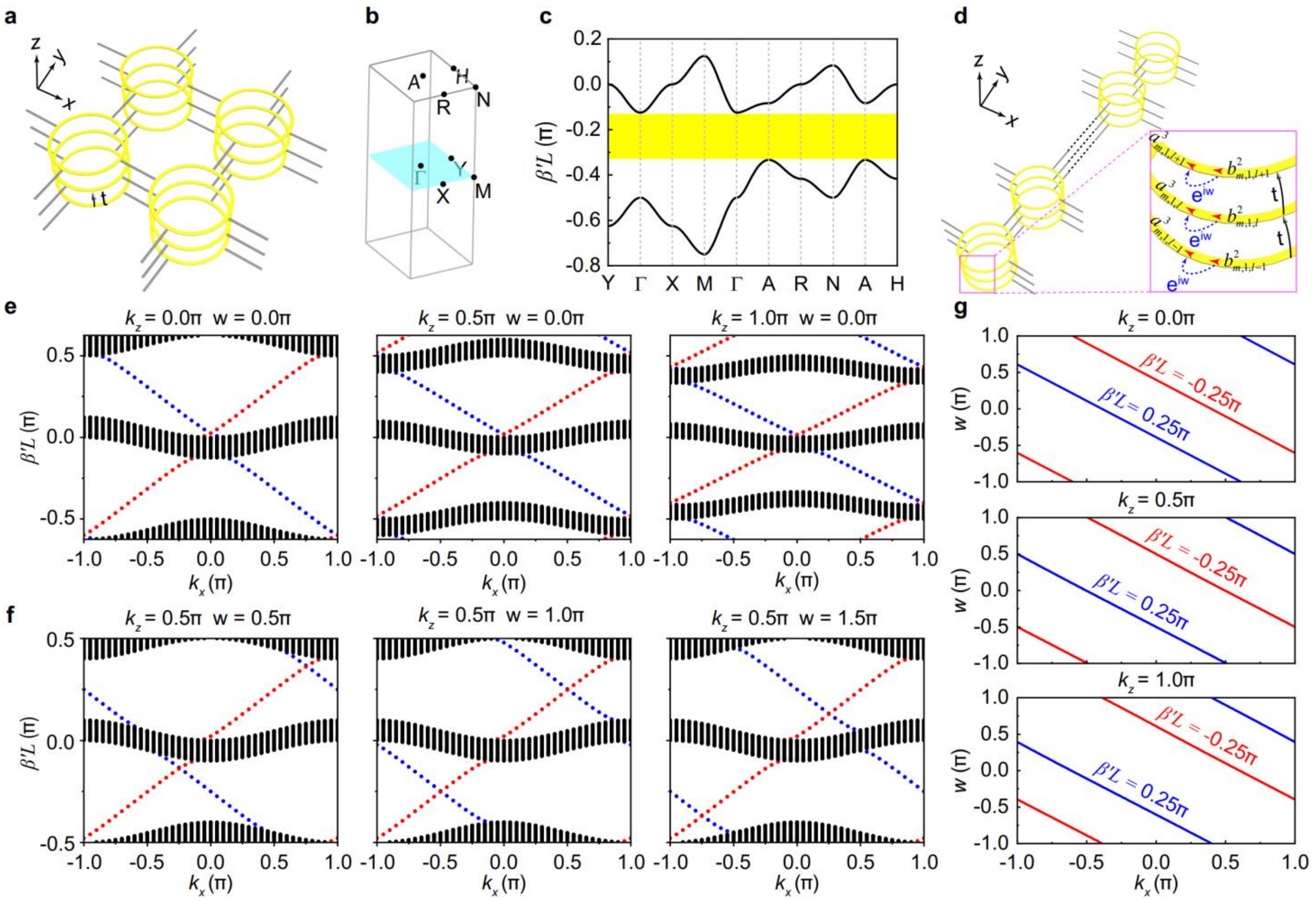

Supplementary figure 5 3D weak topological insulator stacked by layers of 2D QSH insulators with weak interlayer coupling. a, Sketch of 3D structure stacked by layers of the 2D strong coupled ring-waveguides which verified as a QSH insulator in Supplementary Fig. 2. The yellow circles represent the site ring-waveguides and gray lines denote the in-plane couplings between different site ring-waveguides. **b**, The first bulk Brillouin zone of the 3D weak topological insulator. **c**, The band structure simulated along some high-symmetry directions of the Brillouin zone. **d**, A supercell which has $N_y = 20$ sites in the *y* direction with variable phase delays *w* along the lower boundary and periodic in the *x* and *z* directions. **e**, The projected band structures for different k_z with $w = 0$, where the black dots represent bulk states, and the red and blue dots represent surface states on the upper and lower surfaces of the strip geometry, respectively. **f**, The same as **e** but for different *w* with $k_z = 0.5\pi$. **g**, Plots of the boundary angle *w* versus k_x for different k_z. Plots are given for $\beta' L = 0.25\pi$ and $\beta' L = -0.25\pi$, lying respectively, in two different bulk band gaps

Supplementary Fig. 5a gives the sketch of 3D structure stacked by layers of the 2D strong coupled ring-waveguides which verified as an acoustic analog of QSH insulators (which can also be dubbed as anomalous Floquet topological insulator). Here, we assume the site ring-waveguides couple in the *z* direction (same as our experimental setup) with a small coupling coefficient *t* = -0.1, and the in-plane

coupling strength of the ring-waveguides is $\theta = 0.4\pi$. We still adopt the transfer matrix method to analyze this 3D system, assume no pseudospin flipping and only focus on the pseudospin-down component. Similar to 2D system, we use $\left|a_{m,n,l}\right\rangle = \left|a^1_{m,n,l}, a^2_{m,n,l}, a^3_{m,n,l}, a^4_{m,n,l}\right\rangle$ and $\left|b_{m,n,l}\right\rangle = \left|b^1_{m,n,l}, b^2_{m,n,l}, b^3_{m,n,l}, b^4_{m,n,l}\right\rangle$ to represent the wave amplitudes in the site ring-waveguide, with the extra subscript "l" labeling the layer number. The in-plane coupling pattern is the same as the 2D system, i.e., $\left(a^4_{m+1,n,l}, a^2_{m,n,l}\right) = S_x\left(b^1_{m,n,l}, b^3_{m+1,n,l}\right)$ and $\left(a^3_{m,n+1,l}, a^1_{m,n,l}\right) = S_y\left(b^4_{m,n,l}, b^2_{m,n+1,l}\right)$, and the only difference lies in the phase delay along each quarter site ring-waveguide which now depends on k_z, the Bloch wave vector in the z direction. Transformed into the k_z space, we have $\left|b_{m,n,k_z}\right\rangle = e^{i\beta' L(1+2t\cos k_z)}\left|a_{m,n,k_z}\right\rangle$, where L is the propagation length along each quarter site ring-waveguide, and β' is the propagation constant of the waveguide without interlayer coupling[7]. Once again, we refer to $\beta' L$ as the "quasi-energy". For a periodic system, the band structure along some high-symmetry directions of the Brillouin zone (see Supplementary Fig. 5b) is shown in Supplementary Fig. 5c, where we can see the band gap preserves (highlighted with yellow) in the presence of weak interlayer coupling.

To characterize the topological behavior of the band gap, we consider a strip as shown in **d**, which has $N_y = 20$ sites in the y direction and periodic in the x and z directions. Similar to that in Ref. [4], we set the boundary condition in upper boundary as $\left|a^1_{m,N_y,l}\right\rangle = \left|b^4_{m,N_y,l}\right\rangle$ while the lower boundary with variable phase delay w acting as a tunable boundary condition, i.e., $\left|a^3_{m,1,l}\right\rangle = e^{iw}\left|b^2_{m,1,l}\right\rangle$. Thus, we have $S(k_x, w)\left|b\right\rangle = \left|a\right\rangle = e^{-i\beta' L(1+2t\cos k_z)}\left|b\right\rangle$, then the values of $\beta' L(k_x, k_z)$ form a projected w-dependent quasi-energy band structure for the semi-infinite lattice as shown in Supplementary Figs. 5e and 5f. For quasi-energy inside the band gap, only the dispersion curves for edge states localized to the lower edge is affected by the change of w when assuming N_y is large enough such that the upper and lower edge states decouple. Meanwhile, winding w by 2π has the effect of transporting a band of edge states across each quasi-energy gap [4]. It is easy to prove that w (k_x) will come back to itself (modulo 2π) as k_x is advanced over $[0,2\pi]$. Hence if w (k_x) winds once across $[0,2\pi]$ as k_x varies across the Brillouin, there is one band pumping across the quasi-energy gap, and such a quasi-energy gap is topologically nontrivial according to the adiabatic pumping method introduced by Laughlin[8]. As proposed in Ref. [4], the topological boundary invariant n counts the winding number of w (k_x). Obviously, if topological invariant n has non-zero value for the quasi-energy inside a band gap, the band gap is topologically nontrivial; otherwise, it is topologically

trivial. Furthermore, the topological invariant n has a close mathematical relationship with the Chern number of the bulk bands, that is, the Chern number of each band is equal to the difference between the invariant n for the band gaps above and below the band [4,5].

The above discussions have been clearly confirmed by the projected band structures in Supplementary Figs. 5e and 5f and the plots of the boundary angle w versus k_x in Supplementary Fig. 5g. From the projected band structures in Supplementary Fig. 5e, we can observe that the gapless surface states traverse the band gaps independent of k_z. Note that, as we vary the boundary angle w, the angle variable defining the boundary condition at the lower surface, the surface states on the lower surface (blue dots) shift, but still traverse the band gaps, see structure for different w at $k_z = 0.5\pi$ in Supplementary Fig. 5f. The dispersions in supplementary Figs. 5e and 5f also reveal the fact: during one cycle of w, each band of lower surface states winds across the Brillouin zone with the overall effect of pumping one band down across each gap. As proposed in Ref. [4], this is the generic effect of adiabatic pumping on quasi-energy band structures. The data in Supplementary Fig. 5g shows that the w (k_x) has -1 winding number in each gap, which is consistent with the projected band structure in Supplementary Figs. 5e and 5f, where gapless surface states (blue dots) traverse each gap. According to the mathematical relationship between winding number of $w(k_x)$ and topological boundary invariant n discussed above, the invariant of the 3D system is $n = -1$. We have also checked that, the gapless topological surface states also emerge on the y-z surfaces of this 3D structure and no topological surface states emerge on the x-y surfaces of this 3D structure (the data not provided here). The above results confirm that, the 3D structure is a 3D weak topological insulator.

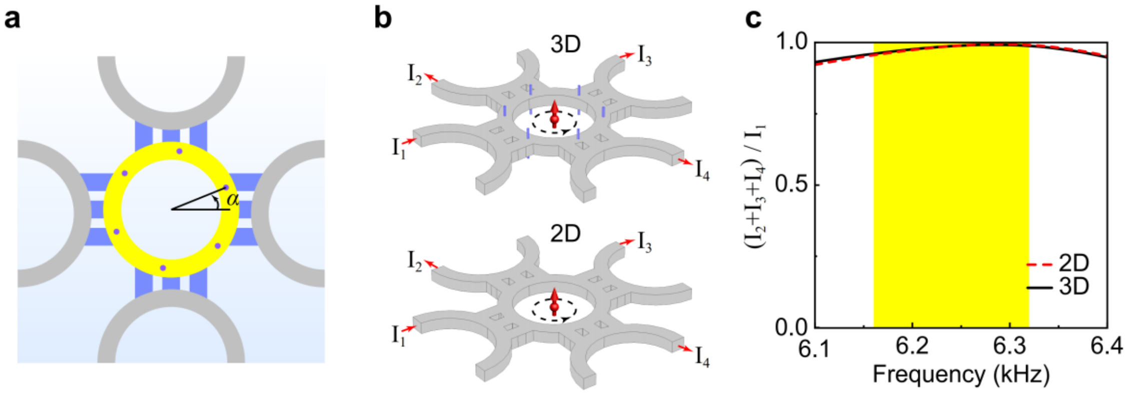

Supplementary figure 6 Design of our 3D ATI. a, Top view of the unit cell. Its in-plane geometry inherits that of the 2D system in supplementary Fig. 1, associated with a constant thickness $h = 10\,\mathrm{mm}$ for 3D realization. The equally-spaced purple dots represent narrow tubes (of radii $r_0 = 1\,\mathrm{mm}$) that couple the site ring-waveguides in the *z* direction, where the angle $\alpha = 22^{\mathrm{o}}$ is optimized to reduce pseudospin flip. The out-of-plane lattice constant of the 3D ATI is $H = 27.5\,\mathrm{mm}$. **b**, Numerical setups for analyzing the coupling ratio of the same pseudospin. Comparing with that of the 2D case (bottom panel), additional periodic boundary condition in the *z* direction is applied to our 3D ATI (top panel). To evaluate the pseudospin preservation, the sound signal is injected from the port 1 of the structure. As such, pseudospin-up modes will be excited dominantly, which leave the structure from the ports 2, 3, 4. The coupling ratio (of the pseudospin-up) is defined as $(\mathrm{I}_2 + \mathrm{I}_3 + \mathrm{I}_4)/\mathrm{I}_1$, where I_1-I_4 are sound energies flowing through the ports 1-4. **c**, Coupling ratio spectra of the 2D and 3D systems. It shows that the coupling ratio of the 3D ATI is almost the same as the 2D system, and approaches 1 within the nontrivial band gap (yellow shadow). In other words, a negligible inter-pseudospin scattering is incurred by the interlayer couplers (i.e., vertical tubes).

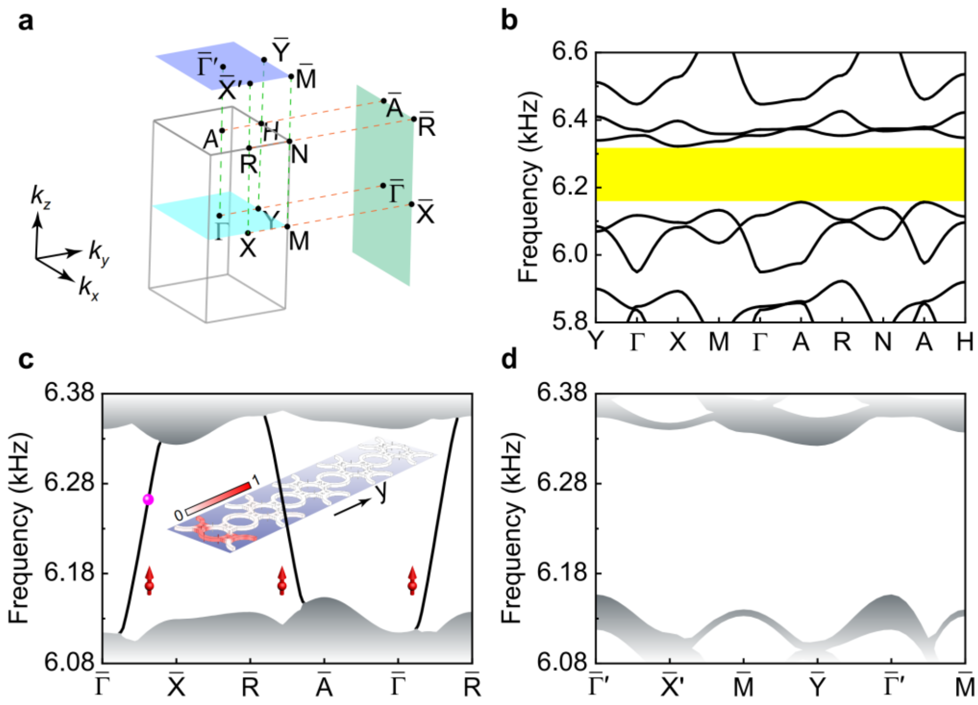

Supplementary figure 7 Bulk topology of the 3D ATI manifested as its surface signatures. a, Bulk Brillouin zone and its surface projections along the k_y and k_z directions. **b**, Bulk band structure simulated along some high-symmetry directions. It exhibits a nontrivial gap ranging from 6.16 to 6.32 kHz (yellow shadow), very close to that of the 2D QSH insulator since the interlayer coupling is weak. **c**, Surface-projected band structure, simulated for a ribbon structure periodic in the *x*-*z* plane but finite along the *y* direction. The black lines indicate gapless topological surface states (plotted only for the surface involved in the inset), and the gray shadows correspond to the projected bulk states. Inset: pressure amplitude distribution exemplified for the surface state labeled with the pink sphere. Similarly, gapless topological surface states emerge on the *y*-*z* surfaces of the 3D ATI. **d**, Surface-projected dispersion simulated for a ribbon periodic in the *x*-*y* plane but finite along the *z* direction, which exhibits no topological surface states on the *x*-*y* surfaces. The simulations in **c** and **d** are consistent with the weak topological indices $(\nu_1\nu_2\nu_3) = (001)$. Physically, the nontrivial topology is inherited from the 2D QSH system since there is no gap closing if one increases gradually the strength of the interlayer coupling from zero to the weak coupling in our system[9].

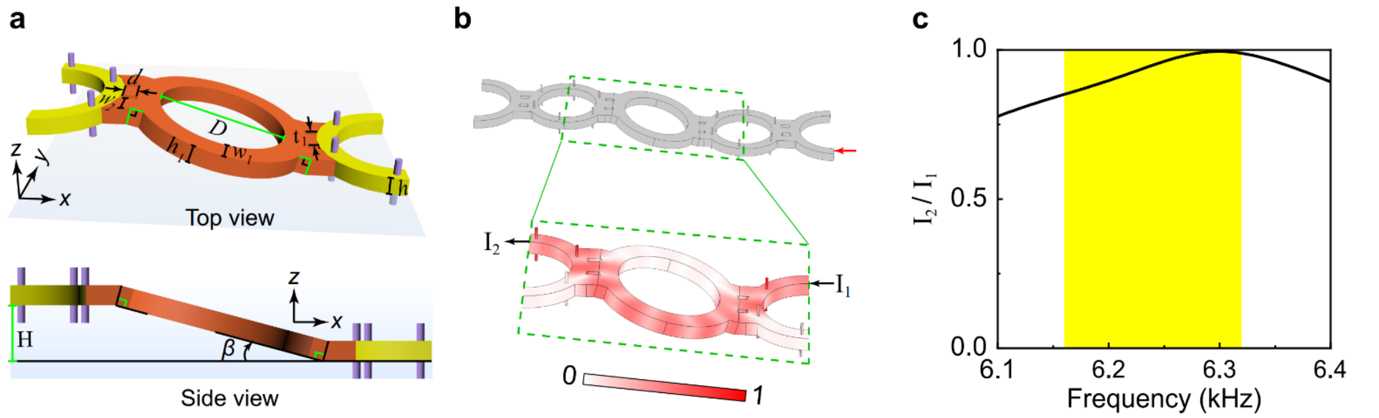

Supplementary figure 8 Design of the tilted interlayer coupler. **a**, Top and side views of the tilted interlayer coupler (orange), which serves as an extra but crucial component for constructing the dislocation. The structural parameters, $t_1 = 19.3$ mm, $w_1 = 14.5$ mm, $w_2 = 14.7$ mm, $d = 8$ mm, $D = 77.3$ mm, $h = 10$ mm, $h_1 = h/\cos\beta$, and $\beta = 15^{\mathrm{o}}$, are optimized to attain a strong coupling but negligible pseudospin flip. **b**, Numerical setup used for analyzing the coupling strength induced by the tilted interlayer coupler, imposed with a periodic boundary condition in the *z* direction. The red arrow indicates the input of the sound signal associated to pseudospin-up. The coupling strength of the tilted interlayer coupler can be characterized by the power transmission $\mathrm{I}_2/\mathrm{I}_1$. Inset: Pressure amplitude distribution exemplified at 6.27 kHz, exhibiting a signature of strong coupling. **c**, Spectrum of the coupling strength, which is larger than 0.85 over the nontrivial gap (yellow shadow).

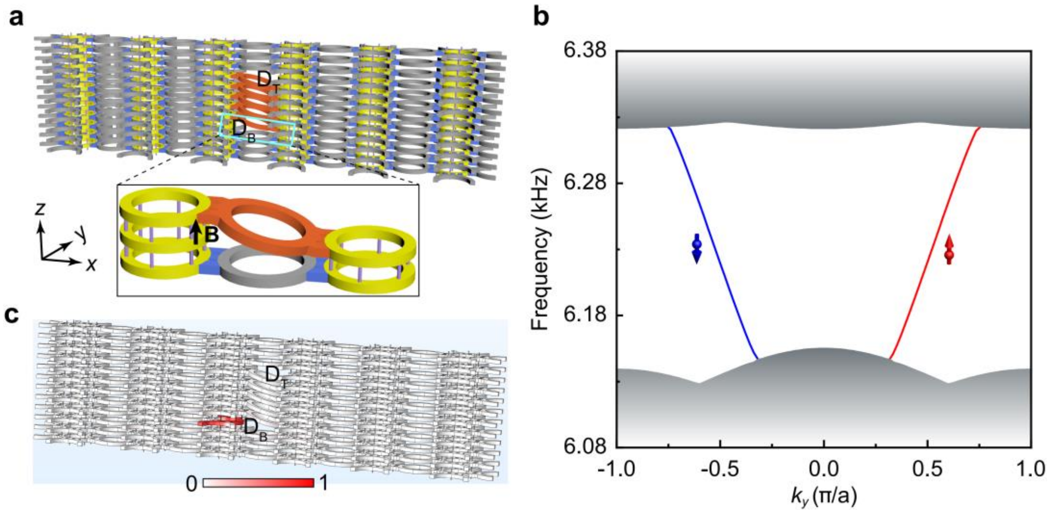

Supplementary figure 9 1D gapless TDMs trapped by edge dislocations. **a**, Supercell structure that contains a pair of edge dislocations with $\mathbf{B} = (0,0,\pm\mathrm{H})$. The supercell has a total size of $6 \times 1 \times 12$ lattice sites in the *x*, *y*, and *z* directions, respectively. By removing 1 interlayer coupler and tilting 5 interlayer couplers (orange), we create two edge dislocations of opposite Burgers vectors, labeled respectively with D_T and D_B. Inset: Zoom-in around the edge dislocation D_B. **b**, Dislocation-projected band structure simulated for the supercell, imposing with Bloch boundary condition in the *y* direction and periodic boundary conditions in the *x*-*z* plane. As expected from the bulk-dislocation correspondence, each edge dislocation supports one pair of helical TDMs. (For clarity, here we plot only the TDMs trapped at the edge dislocation D_B.) **c**, Typical eigenfield distribution (exemplified at 6.27 kHz) for the TDM trapped at D_B.

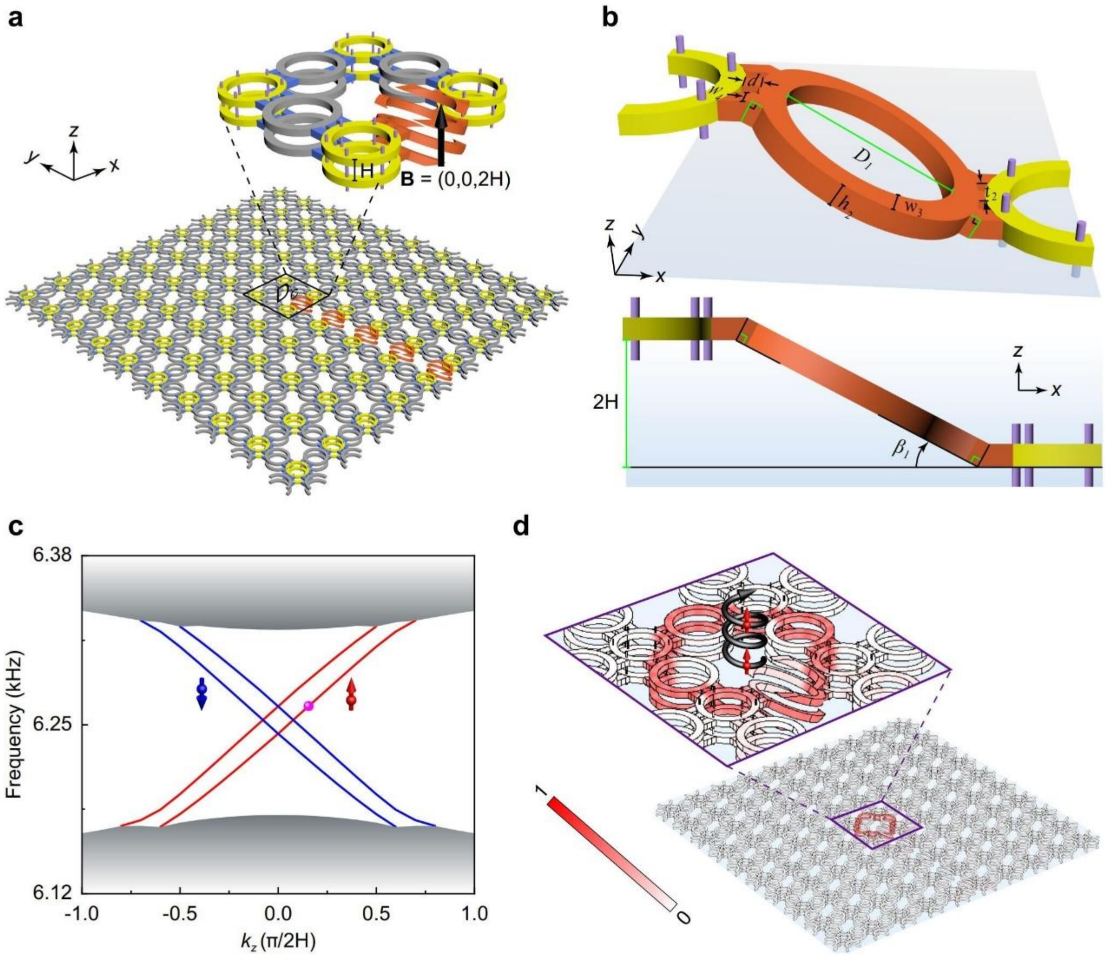

Supplementary figure 10 TDMs trapped by the screw dislocation with a larger Burgers vector. a, Supercell structure that contains a pair of screw dislocations with $\mathrm{B} = (0,0,\pm 2\mathrm{H})$, one at the center and the other at the boundary. The inset shows the fine features around the dislocation in the center (labeled with $\mathrm{D_C}$). **b**, Top and side views of the tilted interlayer coupler (orange), associated with structure parameters: $D_1 = 96.3$ mm , $t_2 = 19.5$ mm , $w_3 = 11.2$ mm , $d = 8.0$ mm , $w_4 = 11.0$ mm , $\beta_1 = 27.6^{\mathrm{o}}$, and $h_2 = h/\cos\beta_1$. **c**, Dislocation-projected band structure simulated for the supercell in **a**, imposing with Bloch boundary condition in the *z* direction while periodic boundary conditions in the *x*-*y* plane. (Here we plot only the TDMs trapped at the supercell center.) As expected from the bulk-dislocation correspondence, each screw dislocation hosts two pairs of helical TDMs (color lines). Note here, unlike the electronic quantum spin Hall system, these two helical TDMs cannot be gapped out with spin preserved interaction. **d**, Pressure amplitude distribution exemplified for the TDM highlighted by the pink sphere in **c**, where the inset enlarges the region around the dislocation.

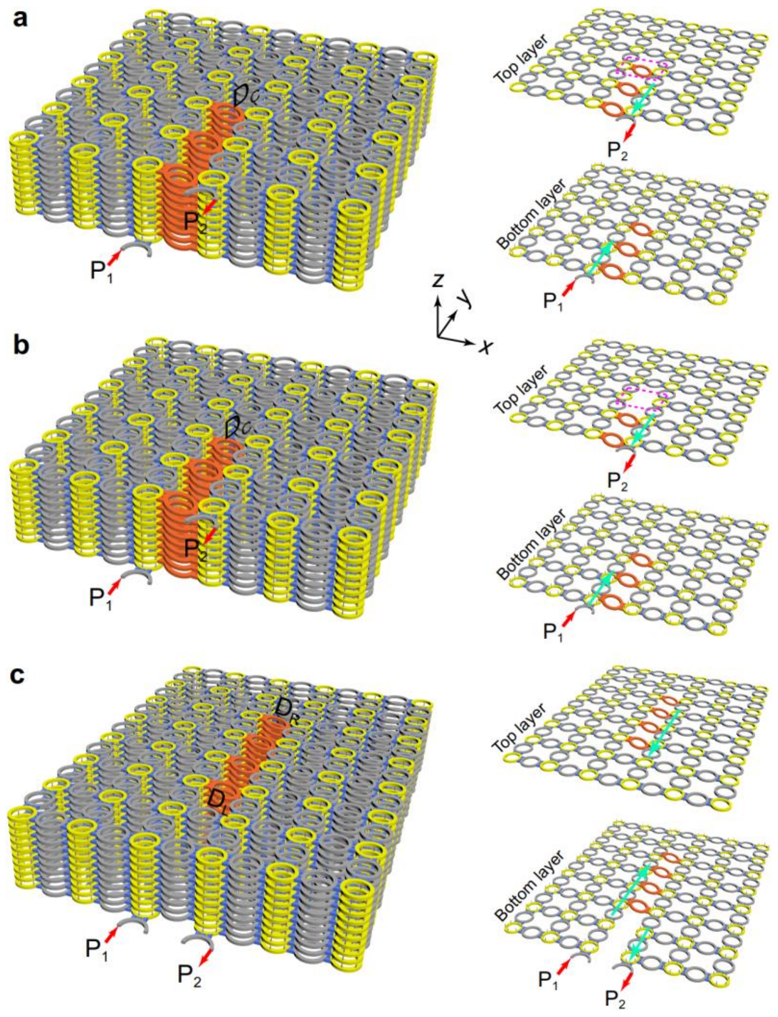

Supplementary figure 11 Structure details of the three dislocation systems discussed in Fig. 3. a, The system considered in Fig. 2e, which hosts a screw dislocation (labeled with D_C) in its interior and two edge dislocations on the top and bottom layers. For clarity, here we provide the structure details for the top and bottom layers. The ports P_1 and P_2, which connect respectively the edge dislocations in the bottom and top layers, are used to import and export sound signals, respectively. The cyan arrows indicate the propagations of the excited TDMs along the edge dislocations on the boundary layers. **b**, The system considered in Fig. 2f. Comparing with **a**, a tilted interlayer coupler on the top layer (see the magenta dashed box) is removed to create a defect in the dislocation path. **c**, The system considered in Fig. 2g. It features a dislocation loop connected with two screw dislocations (labeled with D_R and D_L) in the interior of the sample and two edge dislocations on the top and bottom layers.

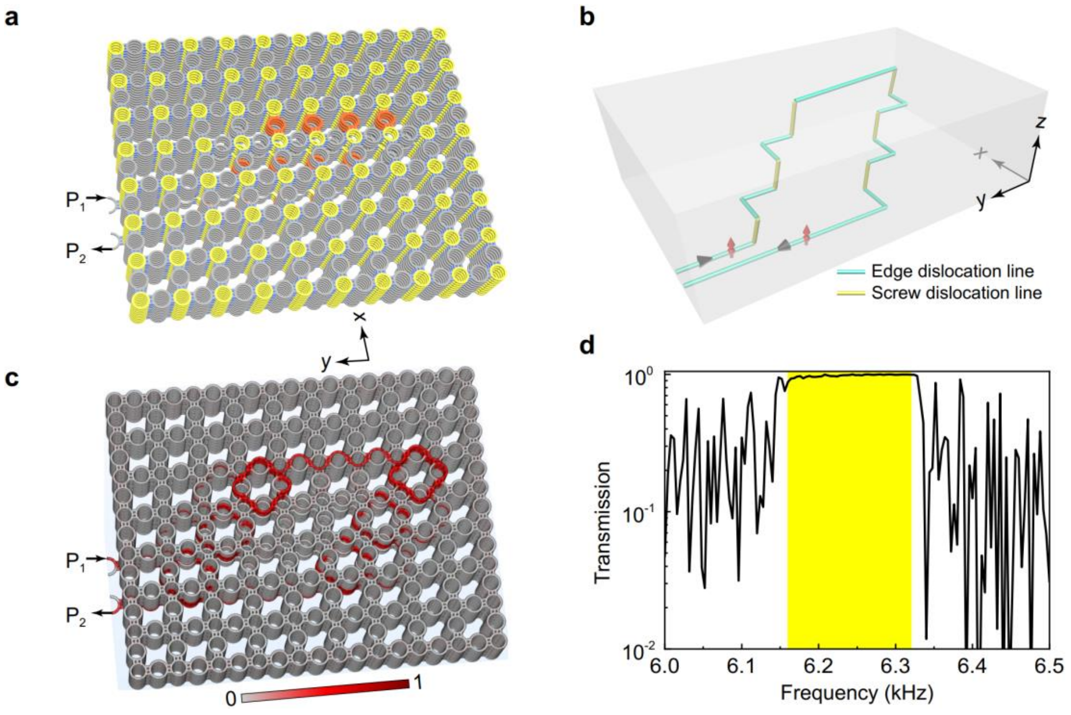

Supplementary figure 12 Unidirectional propagation of the spin-locked TDMs in an arbitrary dislocation path. **a**, A 10-layer 3D ATI with a dislocation path of irregular shape. The sound signal is injected from the port P_1 and exported from the port P_2. **b**, Sketch of the dislocation lines in **a**. **c**, Pressure amplitude distribution simulated at 6.27 kHz. It shows that the (pseudospin-up) TDM propagates smoothly along the prescribed route without visible scattering into the bulk. **d**, Transmission spectrum of the system, which characterizes quantitatively the topological robustness of the TDMs traveling in this complex dislocation path. It shows that nearly perfect transmission occurs within the nontrivial band gap (yellow), while suffering striking reflection beyond that frequency range.

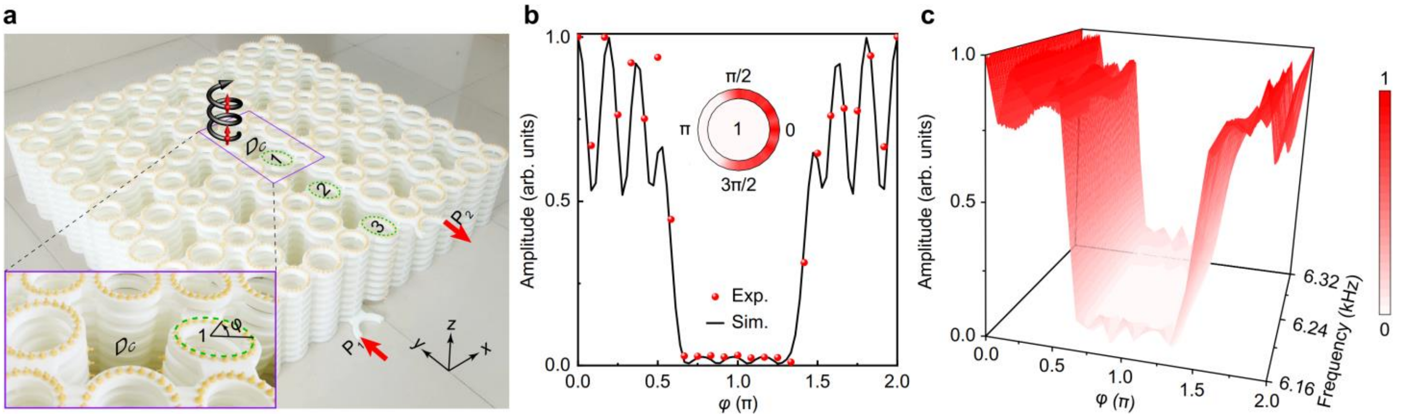

Supplementary figure 13 Experimental evidence for the strong coupling induced by the tilted interlayer coupler. **a**, A photograph of the experimental sample. As mentioned in our main text, pseudospin-up TDMs will be excited for the sound signal injected from the port P_1. The inset amplifies the tilted ring-waveguide 1 around the dislocation D_C, where the angular dependent field profile will be detected hole-by-hole. **b**, Experimentally measured pressure amplitude distribution at 6.27 kHz (red spheres), which reproduces well the simulation result (black line). Inset: A direct visualization of the experimental data inside the ring. The sound energy concentrated in the right half ring shows a direct evidence of the strong coupling induced by the tilted interlayer coupler. (Please pay attention to the coordinate systems defined for supplementary Fig. 8 and Fig. 13.) **c**, Similar to **b**, but plotted from 6.16 kHz to 6.32 kHz, which confirms the strong coupling over the nontrivial bulk gap.

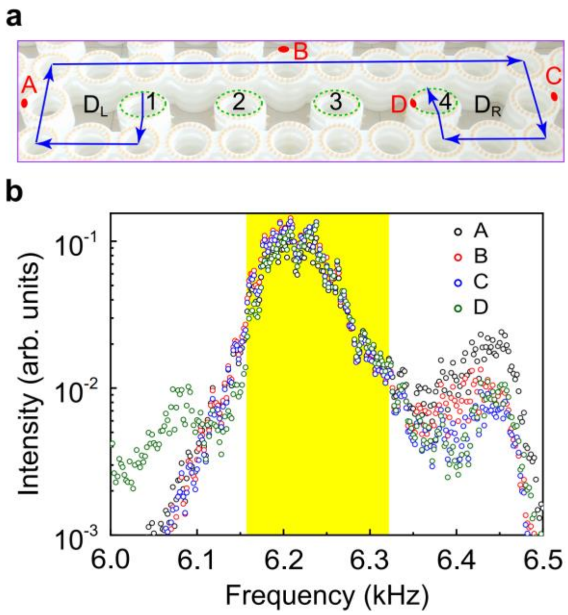

Supplementary figure 14 Intensity spectra along the dislocation loop. a, Zoom-in photograph for the area near the dislocation loop (see Fig. 4a in the main text), which labels four positions (A-D) for sound detection. Again, the blue arrows indicate the propagating directions of the excited TDMs. **b**, Intensity spectra detected at the four positions, where the magnitudes of the cases B-D are compensated by the dissipation factor $e^{-2.1\Delta l}$, with Δl being the corresponding propagation lengths measured from the position A. It shows that the shapes of the spectra within the nontrivial band gap are well-preserved as the sound propagation, markedly different from the data beyond the gap. This spectrum signature serves as a further evidence for the pseudospin-locked unidirectional sound propagation along the dislocation loop.

Reference:

1. Liang, G. Q. & Chong, Y. D. Optical Resonator Analog of a Two-Dimensional Topological Insulator. *Phys. Rev. Lett.* **110**, 203904 (2013).
2. Peng, Y. G. *et al.* Experimental demonstration of anomalous Floquet topological insulator for sound. *Nat. Commun.* **7**, 13368 (2016).
3. Wei, Q., Tian, Y., Zuo, S.-Y., Cheng, Y. & Liu, X.-J. Experimental demonstration of topologically protected efficient sound propagation in an acoustic waveguide network. *Phys. Rev. B* **95**, 094305 (2017).
4. Pasek, M. & Chong, Y. Network models of photonic Floquet topological insulators. *Phys. Rev. B* **89**, 075113 (2014).
5. Rudner, M., Lindner, N., Berg, E. & Levin, M. Anomalous Edge States and the Bulk-Edge

Correspondence for Periodically Driven Two-Dimensional Systems. *Phys. Rev. X* **3**, 031005 (2013).

6. Kitagawa, T., Berg, E., Rudner, M. & Demler, E. Topological characterization of periodically driven quantum systems. *Phys. Rev. B* **82**, 235114 (2010).
7. Rechtsman, M. *et al.* Photonic Floquet topological insulators. *Nature* **496**, 196–200 (2013).
8. Laughlin, R. Phys. Rev. B 23, 5632 (1981).
9. Fu, L. & Kane, C. L. Topological insulators with inversion symmetry. *Phys. Rev. B* **76**, 045302 (2007).
